# Supplementary material for: HIVprotI: an integrated web based platform for prediction and design of HIV proteins inhibitors
Source: J Cheminform. 2018 Mar 9;10:12. doi: 10.1186/s13321-018-0266-y (PMC5845081; doi:10.1186/s13321-018-0266-y)
Supplement: Supplementary file 2 — Additional file 2. Source code of HIVProtI web server. [file 13321_2018_266_MOESM2_ESM.zip › HIVprotI_Source-code/hivprotI/weka-3-6-6/doc/weka/gui/beans/KnowledgeFlowApp.html]

KnowledgeFlowApp


---


|  |  |  |
| --- | --- | --- |
| **Overview** \| **Package** \| **Class** \| **Tree** \| **Deprecated** \| **Index** \| **Help** \| **Weka's home** | |  |
| **PREV CLASS**   **NEXT CLASS** | **FRAMES**    **NO FRAMES**     **All Classes** |  |
| SUMMARY: NESTED \| FIELD \| CONSTR \| METHOD | DETAIL: FIELD \| CONSTR \| METHOD |  |


---


## weka.gui.beans Class KnowledgeFlowApp

```
java.lang.Object
  java.awt.Component
      java.awt.Container
          javax.swing.JComponent
              javax.swing.JPanel
                  weka.gui.beans.KnowledgeFlowApp
```

**All Implemented Interfaces:**: java.awt.image.ImageObserver, java.awt.MenuContainer, java.beans.PropertyChangeListener, java.io.Serializable, java.util.EventListener, javax.accessibility.Accessible

---

``` public class KnowledgeFlowApp extends javax.swing.JPanel implements java.beans.PropertyChangeListener ```

Main GUI class for the KnowledgeFlow. Modifications to allow interoperability
with swt provided by Davide Zerbetto (davide dot zerbetto at eng dot it).

**Since:**
:   1.0

**Version:**
:   $Revision: 7211 $

**Author:**
:   Mark Hall

**See Also:**: `JPanel`, `PropertyChangeListener`, Serialized Form

---

**Nested Class Summary**

**Nested classes/interfaces inherited from class javax.swing.JComponent**

`javax.swing.JComponent.AccessibleJComponent`

**Nested classes/interfaces inherited from class java.awt.Component**

`java.awt.Component.BaselineResizeBehavior`


| **Field Summary** | |
| --- | --- |
| `static java.lang.String` | `FILE_EXTENSION`             the extension for the serialized setups (Java serialization) |
| `static java.lang.String` | `FILE_EXTENSION_XML`             the extension for the serialized setups (Java serialization) |
| `static java.util.Vector` | `s_startupListeners` |

**Fields inherited from class javax.swing.JComponent**

`TOOL_TIP_TEXT_KEY, UNDEFINED_CONDITION, WHEN_ANCESTOR_OF_FOCUSED_COMPONENT, WHEN_FOCUSED, WHEN_IN_FOCUSED_WINDOW`

**Fields inherited from class java.awt.Component**

`BOTTOM_ALIGNMENT, CENTER_ALIGNMENT, LEFT_ALIGNMENT, RIGHT_ALIGNMENT, TOP_ALIGNMENT`

**Fields inherited from interface java.awt.image.ImageObserver**

`ABORT, ALLBITS, ERROR, FRAMEBITS, HEIGHT, PROPERTIES, SOMEBITS, WIDTH`


**Constructor Summary**

`KnowledgeFlowApp(boolean showFileMenu)`
  
          Creates a new `KnowledgeFlowApp` instance.


| **Method Summary** | |
| --- | --- |
| `static void` | `addStartupListener(StartUpListener s)`             Add a listener to be notified when startup is complete |
| `void` | `clearLayout()` |
| `static void` | `createSingleton(java.lang.String[] args)`             Create the singleton instance of the KnowledgeFlow |
| `java.util.Vector` | `getFlow()`             Gets the current flow being edited. |
| `static java.lang.String` | `getGlobalInfo(java.lang.Object tempBean)`             Utility method for grabbing the global info help (if it exists) from an arbitrary object |
| `static KnowledgeFlowApp` | `getSingleton()`             Return the singleton instance of the KnowledgeFlow |
| `void` | `loadInitialLayout(java.lang.String fileName)`             Loads the specified file at input Added by Zerbetto |
| `static void` | `loadProperties()`             Loads KnowledgeFlow properties and any plugins (adds jars to the classpath) |
| `static void` | `main(java.lang.String[] args)`             Main method. |
| `void` | `propertyChange(java.beans.PropertyChangeEvent e)`             Accept property change events |
| `void` | `saveLayout(java.io.OutputStream out)`             Save the knowledge flow into the OutputStream passed at input. |
| `void` | `setEnvironment(Environment env)`             Set the environment variables to use. |
| `void` | `setFlow(java.util.Vector v)`             Set the flow for the KnowledgeFlow to edit. |

**Methods inherited from class javax.swing.JPanel**

`getAccessibleContext, getUI, getUIClassID, setUI, updateUI`

**Methods inherited from class javax.swing.JComponent**

`addAncestorListener, addNotify, addVetoableChangeListener, computeVisibleRect, contains, createToolTip, disable, enable, firePropertyChange, firePropertyChange, firePropertyChange, getActionForKeyStroke, getActionMap, getAlignmentX, getAlignmentY, getAncestorListeners, getAutoscrolls, getBaseline, getBaselineResizeBehavior, getBorder, getBounds, getClientProperty, getComponentPopupMenu, getConditionForKeyStroke, getDebugGraphicsOptions, getDefaultLocale, getFontMetrics, getGraphics, getHeight, getInheritsPopupMenu, getInputMap, getInputMap, getInputVerifier, getInsets, getInsets, getListeners, getLocation, getMaximumSize, getMinimumSize, getNextFocusableComponent, getPopupLocation, getPreferredSize, getRegisteredKeyStrokes, getRootPane, getSize, getToolTipLocation, getToolTipText, getToolTipText, getTopLevelAncestor, getTransferHandler, getVerifyInputWhenFocusTarget, getVetoableChangeListeners, getVisibleRect, getWidth, getX, getY, grabFocus, isDoubleBuffered, isLightweightComponent, isManagingFocus, isOpaque, isOptimizedDrawingEnabled, isPaintingForPrint, isPaintingTile, isRequestFocusEnabled, isValidateRoot, paint, paintImmediately, paintImmediately, print, printAll, putClientProperty, registerKeyboardAction, registerKeyboardAction, removeAncestorListener, removeNotify, removeVetoableChangeListener, repaint, repaint, requestDefaultFocus, requestFocus, requestFocus, requestFocusInWindow, resetKeyboardActions, reshape, revalidate, scrollRectToVisible, setActionMap, setAlignmentX, setAlignmentY, setAutoscrolls, setBackground, setBorder, setComponentPopupMenu, setDebugGraphicsOptions, setDefaultLocale, setDoubleBuffered, setEnabled, setFocusTraversalKeys, setFont, setForeground, setInheritsPopupMenu, setInputMap, setInputVerifier, setMaximumSize, setMinimumSize, setNextFocusableComponent, setOpaque, setPreferredSize, setRequestFocusEnabled, setToolTipText, setTransferHandler, setVerifyInputWhenFocusTarget, setVisible, unregisterKeyboardAction, update`

**Methods inherited from class java.awt.Container**

`add, add, add, add, add, addContainerListener, addPropertyChangeListener, addPropertyChangeListener, applyComponentOrientation, areFocusTraversalKeysSet, countComponents, deliverEvent, doLayout, findComponentAt, findComponentAt, getComponent, getComponentAt, getComponentAt, getComponentCount, getComponents, getComponentZOrder, getContainerListeners, getFocusTraversalKeys, getFocusTraversalPolicy, getLayout, getMousePosition, insets, invalidate, isAncestorOf, isFocusCycleRoot, isFocusCycleRoot, isFocusTraversalPolicyProvider, isFocusTraversalPolicySet, layout, list, list, locate, minimumSize, paintComponents, preferredSize, printComponents, remove, remove, removeAll, removeContainerListener, setComponentZOrder, setFocusCycleRoot, setFocusTraversalPolicy, setFocusTraversalPolicyProvider, setLayout, transferFocusBackward, transferFocusDownCycle, validate`

**Methods inherited from class java.awt.Component**

`action, add, addComponentListener, addFocusListener, addHierarchyBoundsListener, addHierarchyListener, addInputMethodListener, addKeyListener, addMouseListener, addMouseMotionListener, addMouseWheelListener, bounds, checkImage, checkImage, contains, createImage, createImage, createVolatileImage, createVolatileImage, dispatchEvent, enable, enableInputMethods, firePropertyChange, firePropertyChange, firePropertyChange, firePropertyChange, firePropertyChange, getBackground, getBounds, getColorModel, getComponentListeners, getComponentOrientation, getCursor, getDropTarget, getFocusCycleRootAncestor, getFocusListeners, getFocusTraversalKeysEnabled, getFont, getForeground, getGraphicsConfiguration, getHierarchyBoundsListeners, getHierarchyListeners, getIgnoreRepaint, getInputContext, getInputMethodListeners, getInputMethodRequests, getKeyListeners, getLocale, getLocation, getLocationOnScreen, getMouseListeners, getMouseMotionListeners, getMousePosition, getMouseWheelListeners, getName, getParent, getPeer, getPropertyChangeListeners, getPropertyChangeListeners, getSize, getToolkit, getTreeLock, gotFocus, handleEvent, hasFocus, hide, imageUpdate, inside, isBackgroundSet, isCursorSet, isDisplayable, isEnabled, isFocusable, isFocusOwner, isFocusTraversable, isFontSet, isForegroundSet, isLightweight, isMaximumSizeSet, isMinimumSizeSet, isPreferredSizeSet, isShowing, isValid, isVisible, keyDown, keyUp, list, list, list, location, lostFocus, mouseDown, mouseDrag, mouseEnter, mouseExit, mouseMove, mouseUp, move, nextFocus, paintAll, postEvent, prepareImage, prepareImage, remove, removeComponentListener, removeFocusListener, removeHierarchyBoundsListener, removeHierarchyListener, removeInputMethodListener, removeKeyListener, removeMouseListener, removeMouseMotionListener, removeMouseWheelListener, removePropertyChangeListener, removePropertyChangeListener, repaint, repaint, repaint, resize, resize, setBounds, setBounds, setComponentOrientation, setCursor, setDropTarget, setFocusable, setFocusTraversalKeysEnabled, setIgnoreRepaint, setLocale, setLocation, setLocation, setName, setSize, setSize, show, show, size, toString, transferFocus, transferFocusUpCycle`

**Methods inherited from class java.lang.Object**

`equals, getClass, hashCode, notify, notifyAll, wait, wait, wait`

**Field Detail**

### FILE\_EXTENSION

```
public static final java.lang.String FILE_EXTENSION
```

:   the extension for the serialized setups (Java serialization)

    **See Also:**: Constant Field Values

---


### FILE\_EXTENSION\_XML

```
public static final java.lang.String FILE_EXTENSION_XML
```

:   the extension for the serialized setups (Java serialization)

    **See Also:**: Constant Field Values

---


### s\_startupListeners

```
public static java.util.Vector s_startupListeners
```


**Constructor Detail**

### KnowledgeFlowApp

```
public KnowledgeFlowApp(boolean showFileMenu)
```

:   Creates a new `KnowledgeFlowApp` instance.


**Method Detail**

### loadProperties

```
public static void loadProperties()
```

:   Loads KnowledgeFlow properties and any plugins (adds jars to
    the classpath)

---


### setEnvironment

```
public void setEnvironment(Environment env)
```

:   Set the environment variables to use. NOTE: loading a new layout
    resets back to the default set of variables

    :   **Parameters:**: `env` -

---


### clearLayout

```
public void clearLayout()
```

---


### propertyChange

```
public void propertyChange(java.beans.PropertyChangeEvent e)
```

:   Accept property change events

    :   **Specified by:**: `propertyChange` in interface `java.beans.PropertyChangeListener`
    :   **Parameters:**: `e` - a `PropertyChangeEvent` value

---


### setFlow

```
public void setFlow(java.util.Vector v)
             throws java.lang.Exception
```

:   Set the flow for the KnowledgeFlow to edit. Assumes that client
    has loaded a Vector of beans and a Vector of connections. the supplied
    beans and connections are deep-copied via serialization before being
    set in the layout.

    :   **Parameters:**: `v` - a Vector containing a Vector of beans and a Vector of connections **Throws:**: `java.lang.Exception` - if something goes wrong

---


### getFlow

```
public java.util.Vector getFlow()
                         throws java.lang.Exception
```

:   Gets the current flow being edited. The flow is returned as a single
    Vector containing two other Vectors: the beans and the connections.
    These two vectors are deep-copied via serialization before being
    returned.

    :   **Returns:**: the current flow being edited **Throws:**: `java.lang.Exception`

---


### saveLayout

```
public void saveLayout(java.io.OutputStream out)
```

:   Save the knowledge flow into the OutputStream passed at input. Only
    supports saving the layout data (no trained models) to XML.

    :   **Parameters:**: `out` - the output stream to save the layout in

---


### getGlobalInfo

```
public static java.lang.String getGlobalInfo(java.lang.Object tempBean)
```

:   Utility method for grabbing the global info help (if it exists) from
    an arbitrary object

    :   **Parameters:**: `tempBean` - the object to grab global info from **Returns:**: the global help info or null if global info does not exist

---


### createSingleton

```
public static void createSingleton(java.lang.String[] args)
```

:   Create the singleton instance of the KnowledgeFlow

    :   **Parameters:**: `args` - can contain a file argument for loading a flow layout (format: "file=[path to layout file]") Modified by Zerbetto: you can specify the path of a knowledge flow layout file at input

---


### getSingleton

```
public static KnowledgeFlowApp getSingleton()
```

:   Return the singleton instance of the KnowledgeFlow

    :   **Returns:**: the singleton instance

---


### addStartupListener

```
public static void addStartupListener(StartUpListener s)
```

:   Add a listener to be notified when startup is complete

    :   **Parameters:**: `s` - a listener to add

---


### loadInitialLayout

```
public void loadInitialLayout(java.lang.String fileName)
```

:   Loads the specified file at input
    Added by Zerbetto

---


### main

```
public static void main(java.lang.String[] args)
```

:   Main method.

    :   **Parameters:**: `args` - a `String[]` value


---


|  |  |  |
| --- | --- | --- |
| **Overview** \| **Package** \| **Class** \| **Tree** \| **Deprecated** \| **Index** \| **Help** \| **Weka's home** | |  |
| **PREV CLASS**   **NEXT CLASS** | **FRAMES**    **NO FRAMES**     **All Classes** |  |
| SUMMARY: NESTED \| FIELD \| CONSTR \| METHOD | DETAIL: FIELD \| CONSTR \| METHOD |  |


---
